# Impact of Peer-Led Cancer Education Program on Knowledge, Health Beliefs, Practice, and Self-Esteem Among Pairs of Nepalese High-School Students and Their Knowledge-Sharing Partners

**DOI:** 10.3390/healthcare9010064

**Published:** 2021-01-11

**Authors:** Kritika Poudel, Naomi Sumi, Rika Yano

**Affiliations:** 1 Graduate School of Health Sciences, Hokkaido University, Sapporo 060-0812, Japan; kpoudel01@gmail.com; 2 Faculty of Health Sciences, Hokkaido University, Kita 12, Nishi 5, Sapporo 060-0812, Japan; r-yano@med.hokudai.ac.jp

**Keywords:** cancer education program, school-based intervention, students, knowledge-sharing partners, Nepal

## Abstract

Raising cancer awareness among adolescents can increase their confidence in identifying cancer symptoms and develop healthy habits. This study tested the effectiveness of cancer education based on a new model among high schoolers. A non-randomized control group pre-post-test design study was conducted among 313 pairs of adolescent students and their knowledge-sharing partners in Lalitpur, Nepal. A baseline test was conducted before the education program, and it was followed up at two weeks and three months. Results were measured using a chi-square test, binary logistic regression, and a two-way repeated-measures ANOVA. There was a significant interaction effect of intervention and time on students’ knowledge, beliefs, self-esteem, and practice, along with a change in some scores of knowledge-sharing partners. Joint assignment supported the idea of diffusion of information within the family and in the neighborhood. The peer group discussion could encourage active learning and help students to participate visibly in problem-solving and reflecting more sustainably. Time constraints, lack of human resources, and support groups, might limit this program’s usage; however, preparing guidelines, and connecting communities, organizations, hospitals, volunteer health workers, and survivors can help make it more sustainable and approachable.

## 1. Introduction

Every 1 in 6 of the world’s population are adolescents aged 10 to 19, which constitutes around 1.2 billion people. More than 1.1 million adolescents aged 10–19 years lost their lives in 2016, mainly due to preventable or treatable causes [1]. In 2017, the World Health Organization described adolescent health as the range of approaches to preventing, detecting, or treating young people’s health and well-being. During their adolescence phase, young people acquire new habits and behaviors. Alcohol or tobacco use, lack of physical activity, unprotected sex, or exposure to violence can jeopardize current health of adolescents, as well as when they turn into adults and even the health of their future children [1]. Raising cancer awareness among adolescents has the potential to increase their knowledge and confidence about identifying cancer symptoms early and seeking timely medical help in their adolescence and adulthood [2]. Several studies have highlighted the need for a rigorous approach to the development of interventions to increase cancer awareness and help-seeking behavior among adolescents, which might contribute to their own early diagnosis as well as potentially that of friends and relatives, and thereby survival throughout the life course [3,4].

About 6.38 million of total 28.5 million population in Nepal are adolescents aged 10–19 years which constitutes of about 22% of total population [5]. Recent studies have highlighted the concerns regarding sexual and reproductive health, health behaviors among Nepali adolescents and have focused on importance of school-based interventions in addressing them [6,7]; however, there is little understanding of their level of knowledge regarding cancer.

Simply giving information about an association between specific habits and cancer, even if repeated several times, will lead to increased public awareness, and encourage some to make a minimal effort to change their behavior; however, in general the new habit does not persist and continuing and intensifying this approach is not found to be effective [8]. The shift must be made towards active learning which enables students to have a high level of autonomy and self- monitoring, construct new knowledge and enhance critical thinking, and enables long lasting retention of information [9]. Health education is one strategy for implementing health promotion and disease prevention programs. Providing adolescents with information about increased cancer risks associated with certain preventable behaviors are regarded as one way to encourage protective behaviors to provide the foundation for a healthy adulthood [10]. Decision-making during adolescence is highly affected by peer influence [6,7]. Moreover, adolescents also play an important role in increasing communication between cancer and their families [2].

To date, no studies have been conducted in Nepal that paired family members with children to find out information about cancer communication in families. Hence, this study aims to examine the impact of peer-led cancer education program on the knowledge, health beliefs, practice, and self-esteem among students and their knowledge sharing partners. We set the hypothesis that this cancer education approach will increase knowledge, beliefs, practice, and self-esteem among students as compared to traditional lecture methods.

## 2. Materials and Methods

**Figure 1:**
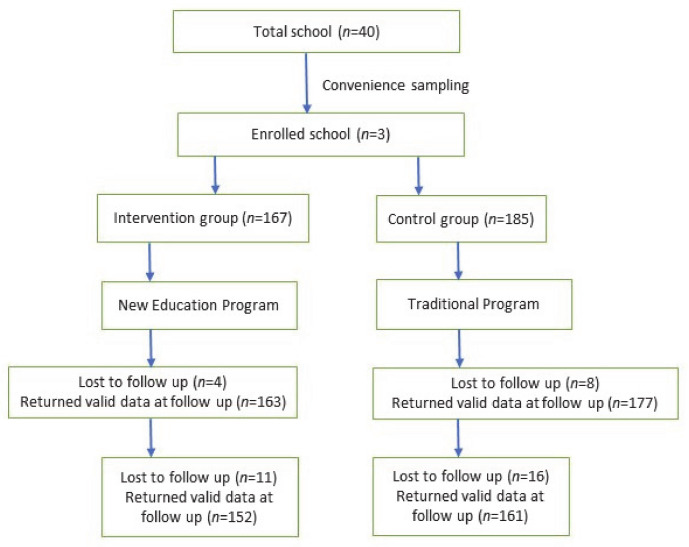
Flowchart of the study.

This study is a quantitative, quasi-experimental design conducted in Lalitpur metropolitan city, Nepal. Lalitpur is considered as one of the major cancers affected districts [11]. Out of total 40 public schools in Lalitpur metropolitan city, 3 schools were selected using convenience sampling. Lottery method was used to separate control and intervention group. High school students studying in same grade were included and were asked to choose one person from their families as a knowledge-sharing partners. Knowledge-sharing partners could be parents or siblings, with whom students could share cancer information. Students were requested to choose same sharing partners throughout the study. After excluding missing data from students and their knowledge-sharing partners, a total of 313 pairs were included in this study. The Figure 1 shows flowchart of the participants.

### 2.1. Cancer Education Program

Cancer education is only present in the curriculum of grade ten under Noncommunicable Diseases in Nepal. While the lesson in the curriculum includes brief information about the risk factors, symptoms, and prevention of cancer, it does not include information on cancer screening, treatment methods, prognosis, cancer awareness activities, and students’ role in cancer prevention. While conducting pilot tests in two schools, we discussed with students about their perception and what they would like to learn about cancer. Based on this, the education program was prepared. In addition to risk factors, symptoms, and cancer prevention, we included global and national epidemiology of cancer, cancer screening and its types, benefits, and barriers to screening and reducing them as a student. While traditional lecture methods have overtaken teaching learning methods in Nepal [12], to promote active learning, a new education package was developed. The general instructive objective of this new cancer education program was to identify and increase health promoting behaviors among adolescents. The specific behavioral objectives were as follows: (i) To describe and discuss overall cancer knowledge with other students using active learning approach and summarize it. (ii) To engage students in group discussion and produce a poster. (iii) To analyze the importance of healthy behavior, share with partners and adopt healthy habits. The two steps of new cancer education program were peer leader training and peer-led cancer education.

#### 2.1.1. Peer Leader Training

In intervention group, peer leaders were selected voluntarily. Peer leaders were the students who were willing to receive training and teach their friends about cancer. Three training sessions of 45 min each were conducted during peer leader training based on the four subscales of the Health Belief Model (perceived susceptibility, severity, benefits, and barriers). The Health Belief Model is one of the popular models in health education which focuses on health behaviors and the possible reasons for non-compliance with recommended health actions. An individual’s likelihood to prevent or detect disease is determined by several factors: perceived susceptibility to the health condition, perceived severity of the health threat, perceived benefits of performing the healthy behavior, and perceived barriers to accomplishing this behavior [13]. The baseline test of peer leaders in intervention group was performed before training.

#### 2.1.2. Peer-Led Cancer Education

**Figure A1:**
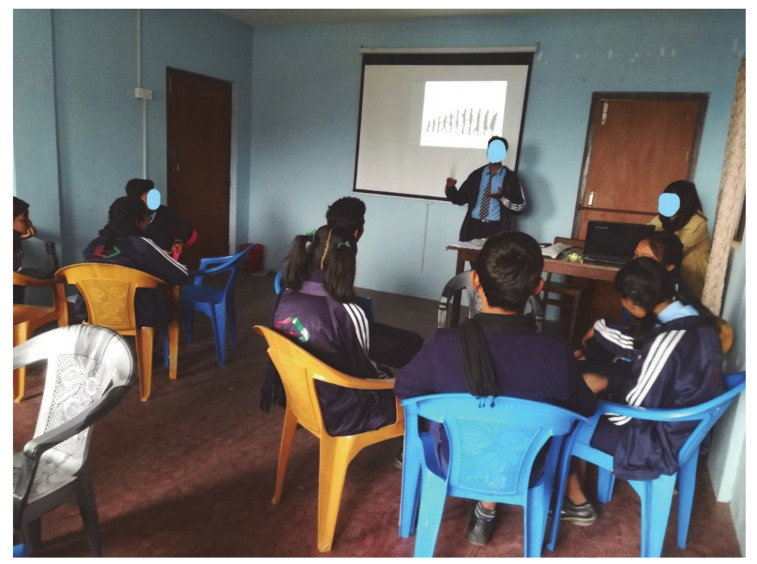
The education program in intervention group.

This step was based on three sessions. The first approach was from peer leaders to students while the session was susceptibility, severity, benefits, and barriers. In this session, peer leaders conducted class on epidemiology, risk, symptoms, prevention, screening, and barriers to screening in Nepal to other students. Classes were conducted using power point slides, quizzes, and group discussion. The second approach was from students to students and the session was based on critical thinking. In this session, students discussed about cancer, formed groups, and engaged in problem findings. The group discussion was based on theme question, “How is cancer present around you?” The third approach was from students to sharing partners and session was based on diffusion of information as per Learning Partner Model, where students completed assignment with their knowledge-sharing partners. In the control group, the researcher conducted a cancer class with same contents but using the traditional lecture method. An immediate post-test was conducted after two week of the education program in both control and intervention groups, along with their knowledge-sharing partners. A second post-test was conducted after three month to check knowledge retention and initiation of healthy practices. Figure A1 shows the glimpse of education intervention on students.

### 2.2. Data Collection Tools

The self- administered questionnaire was used to assess the knowledge of cancer among high school students and their knowledge-sharing partners. Demographic, socio-personal questions were included. The questions about cancer awareness were derived from Cancer Awareness Measures [14], whereas the questions related to health beliefs were prepared using the Health Belief Model. The health belief subscales score was calculated based on a 1–4 Likert scale (4: strongly agree, 3: agree, 2: disagree, 1: strongly disagree). The perceived susceptibility, severity, and barriers consisted of 4 items, and perceived benefits comprised of 6 items. The Rosenberg Self Esteem Scale was used to measure both positive and negative feelings about they felt about themselves [15]. Questions about health-promoting activities based on extensive literature reviews were added. The cancer education content was developed by an extensive literature review, and consultations with medical doctor experts in cancer medical education. Data collection was performed between May and September 2019. The participants recruitment was started in mid-May. The pre-test was conducted in late- May. The intervention program was conducted in early June and it was followed by posttest 1 after two week. The second follow-up was conducted in mid-September.

Questionnaires were translated into Nepali and then reverse-translated into English by experts to ensure retention of same concepts. Content validity was established by an extensive literature review, and consultations with a research advisor and medical doctors. The cancer education content, developed by researchers, was checked and confirmed by a clinician specializing in medical education in Japan. Pilot test was conducted at two different schools of Lalitpur prior to the study. 

#### 2.2.1. Statistical Analyses

Chi-square test was used to compare participants’ demographic characteristics and outcomes at baseline, 2-week and 3-month between the control and intervention group. A logistic regression was conducted to assess the size of change on healthy practice between baseline and three month. For the logistic regression analysis, baseline was coded as “0” and three month was coded as “1”. A t-test was used for continuous variables and a two-way repeated measures ANOVA were used to examine the effects of the intervention and time on outcome variables. The level of significance was set at 0.05. All analyses were performed using the IBM SPSS Statistics for Windows software version 22.0 (IBM Corp., Armonk, NY, USA).

#### 2.2.2. Compliance with Ethical Standards

Ethical approval: This study was approved by the Hokkaido University, Japan, and the Nepal Health Research Council (2805).

Informed consent: Informed consent was obtained from all students and their knowledge-sharing partners in both groups.

## 3. Results

A total of 313 students and their knowledge-sharing partners were included in this study. The response rate of students was 91.01% and their knowledge-sharing partners was 87.02%.

### 3.1. Socio-Personal Information

The median age of the students was 14. There was no difference observed between the responses of the two student groups on the topics: sex, talks on cancer, importance of cancer talks, and wish to undergo cancer screening. The Table 1 presents the socio-personal information of students. The majority of knowledge-sharing partners were parents (71.9%) followed by elder siblings (28.1%). Mothers (46.3%) were the most common knowledge-sharing partners. There was no statistically significant difference between sex (*p* = 0.871), family history of chronic illness (*p* = 0.298), importance of cancer talks within the family (*p* = 0.490), history of cancer classes (*p* = 0.303), and wish to undergo cancer screening (*p* = 0.087) in the control and intervention group. 

### 3.2. Knowledge, Health Beliefs and Self-Esteem

**Figure 2:**
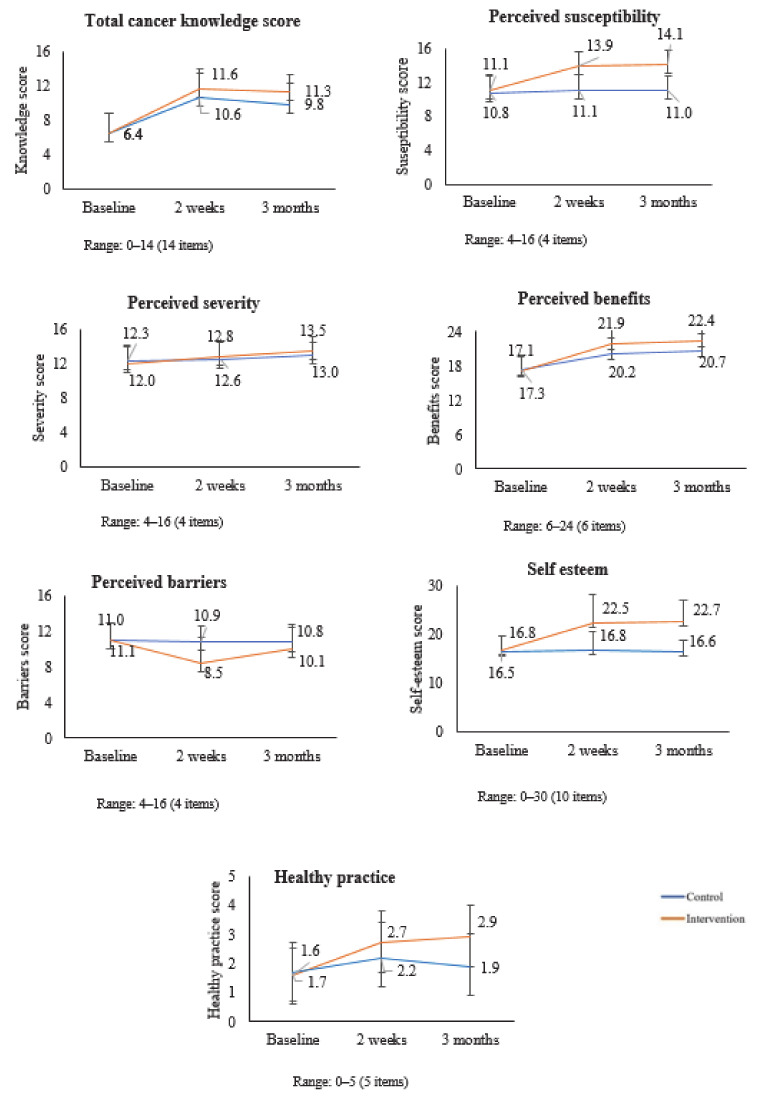
Changes in Knowledge, Total Health Belief Subscale Scores, Self- esteem, and Healthy Practice of Students over Time.

The *t*-test showed that the baseline score of knowledge, perceived susceptibility, perceived severity, perceived benefits, perceived barriers, self-esteem, and health promotion of control, and intervention in students’ group was statistically insignificant. The Figure 2 shows the changes in knowledge, total health belief subscale scores, self-esteem, and healthy practice of students over time. There was a change in the scores of both students’ groups during the two-week and three-month follow-up.

**Figure 3:**
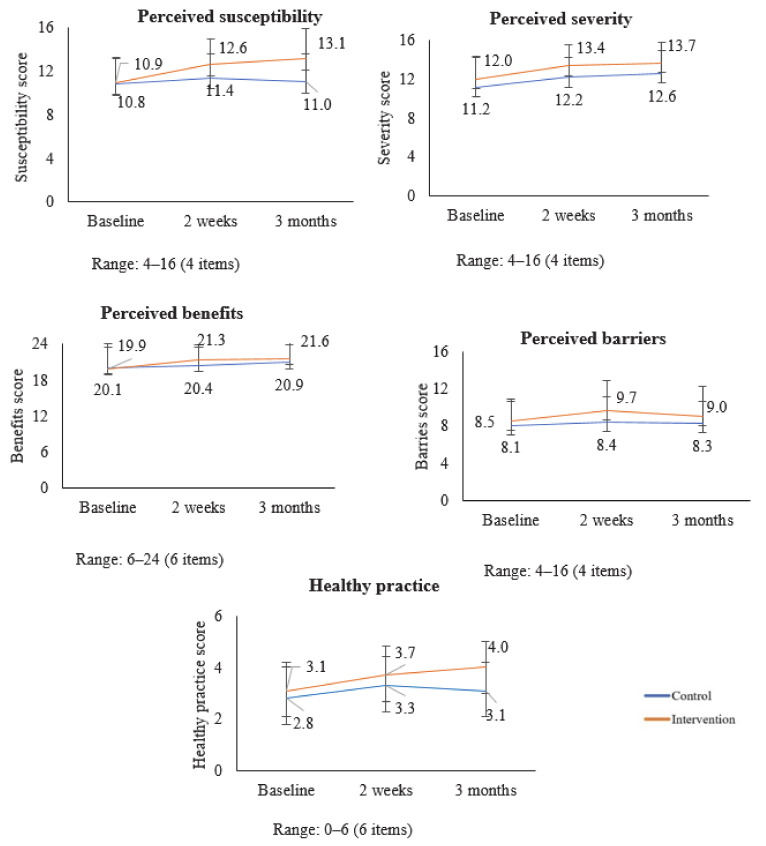
Changes in Total Health Belief Subscale Scores, and Healthy Practice of Knowledge-sharing Partners over Time.

The Figure 3 shows the changes in healthy belief subscale scores and self-reported healthy practice among knowledge-sharing partners. There was an increase in perceived susceptibility, severity, and benefits in both groups. There was a decrease in perceived barriers in both groups. The self-reported healthy practice increased in the intervention group while it decreased in the control group.

A two-way repeated measures ANOVA showed that there was an effect of intervention, time, and interaction in all scores. The Table 2 shows the effects of time and interaction on knowledge, health beliefs subscales, self-esteem, and healthy practice in students. There was a significant difference in the main effect of time on students suggesting that there were changes in student’s scores across three different time periods. The *p*-value showing interaction effect (intervention × time), indicated that there are significant differences between the variable scores of the control and intervention student’s groups over time and treatment except on perceived severity. The effect size (η_p_^2^) showed that the effect of time was stronger than the interaction effect (intervention and time). 

The new cancer education program included cancer communication between students and knowledge-sharing partners through a joint assignment. The t-test showed that the baseline score of perceived severity between the two partners’ groups was statistically significant. A two-way repeated measures ANOVA showed that there was an effect of intervention, time, and interaction in some scores of knowledge-sharing partners. Further information is presented in Table 2.

### 3.3. Healthy Practice

The habit of exercising, sleeping for at least seven hours, consuming more than four to five servings of green vegetables, fiber, cereals per week, cancer talk, and regulating salt intake was considered as healthy practices among students. The Table 3 shows the logistic regression on the change in health behaviors in three-month as compared to those at baseline. In the intervention groups, the odds ratio (OR) for the cancer talk with others was 19.58 in three-month follow-up as compared with baseline (95% CI, 10.50–36.52). Similarly, there was a change in exercise habit (OR: 3.16; 95% CI, 1.97–5.06), sleeping time (OR: 1.71; 95% CI, 1.04–2.82), and healthy diet consumption (OR: 2.76; 95% CI, 1.66–4.60) during three-months follow up. There was a change in the sleeping time (OR: 0.62; 95% CI, 0.40–0.98), healthy diet consumption (OR: 4.50; 95% CI, 2.76–7.35) in the control group as compared to the baseline.

Avoiding smoking and alcohol consumption, exercise, proper consumption of healthy food, keeping a check on salt intake, and cancer screening tests were considered healthy practices among knowledge-sharing partners. The Table 4 shows that in the knowledge sharing partners of intervention groups, the odds ratio (OR) for the non-alcohol habit was 2.27 in three-month follow-up as compared with baseline (95% CI, 1.31–3.94). Similarly, there was a change in exercise habit (OR: 5.33; 95% CI, 3.11–9.15), healthy diet (OR: 3.24; 95% CI, 1.73–6.08), and approach for cancer screening (OR: 4.06; 95% CI, 1.79–9.24) during three-month follow up. There was a change in the healthy diet consumption in the control group as compared to the baseline (OR: 2.96; 95% CI, 1.72–5.09). This suggests that cancer communication was more common in the intervention group, which could connect knowledge-sharing partners in health promotion. Detailed information is presented in Table 4.

## 4. Discussion

Although several studies have been conducted in Nepal to explore cancer awareness among adults, little is known about the impact of cancer communication on adolescents and their knowledge-sharing partners. Furthermore, this is the first study conducted in Nepal, pairing adolescents with their knowledge-sharing partners, and showing the importance of cancer talk among family members. 

This study was conducted in Lalitpur, Nepal. In our study, 51.4% of students were female, which was slightly more than the national census of Lalitpur metropolitan city, (49.1%). The literacy rate among youth (15–24 years) in Nepal was 89.95%, while the literacy rates of males and females between the age of 15 and 25 were 88.2% and 76.7%, respectively [16]. There was no significant difference between the knowledge and gender of students in both groups. As all the students were of the same age and grade, the relationship between these and dependent variables were not checked.

### 4.1. Students

The general objective of the new program was to create awareness among students and encourage communication between adolescents and their families about cancer. The main purpose of specific behavioral objectives was to encourage students to take part individually and in groups through different tasks. To achieve this goal, our program engaged students in three different sessions based on our new model which supports Health belief model. The joint assignment activities and involvement of knowledge-sharing partners in this study was based on Learning Partner Model. Several interventions based on the model have been effective compared to studies without any model [17,18,19].

Previous studies conducted in Nepal have shown less cancer awareness among adolescents [20,21]. Our results showed that there was an increase in knowledge, health beliefs, self-esteem, health-promoting activities as compared to baseline tests among students in intervention group. The intervention had a positive effect on exercise habits, and cancer talking behaviors during the three-month follow-up. A Chinese study showed the effectiveness of health education intervention in increasing physical activity and health behaviors among students, suggesting the need to include health education in the curriculum [22]. As the habits developed during adolescence shape the health in adulthood, there is need to increase cancer awareness among Nepali from their early school age.

The theory of planned behavior stated that intention toward behavior, subjective norms, and perceived behavioral control together shape an individual’s behavioral intentions and behaviors [23]. There was a slight change in the health promotion and cancer screening approach of the partners in the intervention group. It is obvious that more information will lead to increased public awareness and will encourage even the minimal effort to change their behaviors; however, the new habit tends to not persist [8]. Students were directly involved in the teaching-learning process, which helped them to start and continue healthy behaviors even after completion of education programs. Knowledge is the first key element in developing healthy behavior. The most useful source of information is a school- based program. School-based health promotion can be particularly valuable in developing countries facing the challenges of low health literacy and high disease burden [7,24]. 

A significant interaction between intervention and time was observed in this study. There was a substantial main effect of time on both groups, increasing all dependent variable scores across the three time periods. The main effect comparing the two types of intervention was significant (*p* < 0.001), suggesting a difference in the two program approaches. This result suggested that this new program was more effective than traditional lecture methods in the classes. This method could be of tremendous assistance to the students who are still deprived of proper educational access, ample facilities of books and efficient teachers [25]. 

Several studies have supported the idea that adolescents are more likely to modify their behaviors and attitudes if they receive health messages from peers who face similar concerns and pressure [26,27,28]. A Nepali study showed a significant positive impact of a peer-led intervention on increasing self-efficacy and reducing potential risk behaviors related to sexually transmitted diseases among students [29]. Another study showed the efficacy of the classroom-based intervention for improving social-behavioral and positive aspects of well-being indicators among subgroups of children exposed to armed conflict in a low-income country [30]. As peers play an important role in the psychological development of most adolescents, peer education is considered a health promotion strategy in adolescents [31,32]. 

A study found that using video format was more effective than traditional methods of health education which resulted in short-term knowledge but offered no advantages in improving long-term knowledge retention [19]. Studies focused on the use of e-learning for medical education in low- and middle-income countries showed limitations in reaching its full potential due to restricted financial resources and sustainability [33]. As peer learning does not need any this concern needs to be addressed as the effectiveness and long-term impact of health messages ultimately depend on how well the end users can identify with the content that is presented to them.

Our study showed a decrease in knowledge scores compared to the first follow-up. These findings are like another study where there was a decline in knowledge in the three-month follow-up group compared to the immediate group [34]. Although knowledge can decrease with time, it is important to maintain healthy beliefs and practice behaviors.

Our study showed that school- based programs are effective in addressing knowledge of students. During the baseline test, approximately 11% were aware that lung cancer was not the most common cancer among Nepali women. This might be due to a lack of information about other types of cancer. Although the government and some organizations are conducting various programs to make people aware of different types of cancers, lung cancer is most well-known in Nepal due to which information about other types of cancers might have been overshadowed [35].

Our study showed that students in both groups agreed that smoking cessation is a preventive measure of cancer. A Nepali study highlighted that students were aware of smoking and its relation to cancer. It further showed that friends were the most influential factor for smoking among adolescents. The fact that peers can influence smoking habits highlighted the importance of peer education in getting rid of the habit [36].

The baseline test showed that there was no difference in knowledge, health beliefs, self-esteem, and healthy practice of students in both groups. The increase in knowledge, health beliefs, self-esteem, and health-promoting activities among students in traditional lecture groups was similar to that of the intervention group. However, the students in intervention group were able to retain it for longer time in terms of cancer communication. The logistic regression showed that students in the intervention group increased their exercise habit, sleeping time, healthy diet consumption and cancer communication with others during three-month follow-up. A study conducted among students in Northern Lima and Callao has also shown an increased tendency to consume vegetables in the intervention group after the education program [37].

The logistic regression of knowledge-sharing partners also showed significant changes in exercise and healthy habits during three-month follow-up. A school-based intervention in Italy has shown a positive effect of an intervention on children’s food habits and has encouraged the role of school-family collaboration for healthy lifestyles [38]. The new program tried to support students as an individual and as a team to provide them with opportunities to learn both in and out of classroom-based on a shared developmental framework uniting with families and communities as a whole. Studies have suggested that students are more likely to attend school and get attached to learning, when they have strong, trusting connections with adults [39]. Active parental involvement is important for effective school-based health promotion interventions [40].

There was no difference in self-esteem between boys and girls during the baseline test in our study. This might be because adolescent boys and girls are getting equal opportunities in recent years. This finding is in contrast with a Pakistani and Bulgarian study where boys showed higher self-esteem level than girls [41,42]. Another Nepali study conducted among nursing students showed that low self-esteem was due to stress in the academic environment [43]. However, for both genders, self-esteem is relatively high in childhood, drops during adolescence, and rises gradually throughout adulthood before it tends to decline in old age [44].

### 4.2. Knowledge-Sharing Partners

The majority of knowledge-sharing partners were mothers, followed by fathers, sisters, and brothers. During the baseline test, about 3.9% of the partners self-reported to have screened for cancer. This finding is less than another Nepali study in which 15% of participants had taken the cervical cancer screening test. Less screening practice was related to lack of knowledge and awareness about screening in communities, cultural norms, fear of cancer, nonexistence of laboratories in rural areas, and embarrassment [45]. Knowledge-sharing partners in the intervention group had talked more about cancer than in the control group during the baseline test. Many variables such as interests, awareness, education, socioeconomic factors, culture, and family practices might have played a role in increasing talks with other.

The self- reported screening approach increased to 10.2% after the cancer education program during the three-month follow up. The increment was seen more in the intervention group than in the control group. This showed that cancer communication among students and sharing partners might have played a key role and encouraged them to undergo screening tests. Health communication contributes to health promotion by providing health-related information and influences the health behaviors of individuals and communities [3,4]. 

The repeated measures ANOVA also showed some impacts of the new cancer education program on knowledge-sharing partners. Although there was no effect of interaction between intervention and time on perceived severity, benefits, and barriers, the main effect of time was seen on health promotion, and health belief subscales. As our main targets were not knowledge-sharing partners, various factors, such as culture, socioeconomic factors, family practices, and current trends, might affect the knowledge, decision and health behavior of adults. Hence, efforts should be made to involve family-based approaches in health promotion. A study showed that family-based approaches in health promotion for cardiovascular diseases have beneficial effects [46]. Our previous study have also supported communication with mothers as strong predictors of knowledge among students [4]. Our findings support the idea of diffusion of innovation which occurs through different communication channels over time among the members of family and community [47]. The students might have acted as innovators. When students shared information with their families, the majority of participants adopted the idea in the intervention group. However, there might be a gradual drop over time. As this study was limited to a three-month follow up, it would be interesting to observe the long-term, in six months, or a year. By identifying the characteristics of people in adopting new habits, researchers can plan more effectively and implement strategies accordingly [37]. The Ministry of Health and Population in Nepal started implementing “One health worker per school policy” at private schools since 2017. Later, the program expanded to public schools, and since then, there is one health worker, mostly nurse, in each school throughout the province to improve health outcomes of children. Hence, these school-based nurses can act as facilitator to promote peer training in school students and connect with local stakeholders through school organization to address community health issues [32].

It was observed that during second follow up, perceived severity had increased in both students and knowledge-sharing partners. However, the two-way repeated measures ANOVA did not show significant interaction effect of time and intervention on the students. This might be associated with the increasing risks of other epidemics as our second follow up was carried out during August–September when Nepal was having dengue outbreak, reaching 68 out of 77 districts, affecting 16,000 lives [48].

The students could retain their knowledge, beliefs, and initiate new habits, which might be because of their self-engagement in the discussion along with the communication and support from family members. This could indicate the chain of communications among family members in protecting and promoting family health. The learning partner model used in this study possibly enhanced the diffusion of health information to friends or family members of the individuals attending the cancer education sessions [49].

One of the common limitations was the study duration of this study. The non- response rate of sharing partners during three-month follow-up was high. Hence, we excluded data of participants who did not submit questionnaire of their sharing partners or vice versa. The behavioral outcomes of students and their knowledge-sharing partners were measured by the self-reported questionnaires, which could have led to recall bias. In the school-based cancer education program, we used pre- post- test design, so it cannot be presumed to illustrate cause and effect as participants’ knowledge on the cancer topic may have changed as a result of other factors. 

## 5. Conclusions

This study showed that the new cancer education program has the potential to be a beneficial method in Nepali classroom settings to encourage students in active learning and participate visibly in problem-solving and reflecting more sustainably. Using peer-learning strategies does not require abandoning the lecture format; adding small active learning strategies can make the lecture more effective for student learning inside classroom settings and it is also helpful in large classrooms. Health communication with parents, neighbors, or communities was helpful in the dissemination of information about cancer in this study. However, the school-based interventions alone are insufficient to improve adolescent health behaviors. Interventions incorporating community and family-based approaches using local support systems can be effective to reduce the social barriers for behavior change among the schoolchildren and helpful in reinforcing adolescent’s self- perception on harmful behaviors. Time constraints, lack of manpower and support groups might limit the use of this new program in Nepal. However, connecting communities, organizations, volunteer health workers, hospitals and survivors in this new education program can help in making it more sustainable and approachable.

## Figures and Tables

**Table 1:** Socio-personal information of students.

		Intervention (*N* = 152)	Control (*N* = 161)	
Variables	Category	*N* (%)	*N* (%)	*p*-Value
Students				
Sex	Male	70 (46.1)	82 (50.9)	0.388
	Female	82 (53.9)	79 (49.1)	
Members in health field	No	132 (86.8)	140 (87.0)	0.976
	Yes	20 (13.2)	21 (13.0)	
Importance of cancer talk	Very important	20 (13.2)	34 (21.2)	0.093
	Somehow important	121 (79.6)	111 (68.9)	
	Not so important	11 (7.2)	16 (9.9)	
Talked about cancer before	No	106 (69.7)	113 (70.2)	0.931
	Yes	46 (30.3)	48 (29.8)	
Wish to take cancer screening	No	40 (26.3)	48 (29.8)	0.573
	Yes	112 (73.7)	113 (70.2)	
Knowledge-sharing partners				
Relationship	Mother	67 (44.1)	79 (49.1)	0.335
	Father	37 (24.3)	42 (26.1)	
	Elder sister	27 (17.8)	22 (13.7)	
	Elder brother	21 (13.8)	18 (11.2)	
Family history of chronic illness	No	137 (90.1)	139 (86.3)	0.298
	Yes	15 (9.9)	22 (13.7)	
Importance of cancer talks within family	Not important	14 (9.2)	17 (10.6)	0.490
	Somewhat important	105 (69.1)	101 (62.7)	
	Very important	33 (21.7)	43 (26.7)	

Chi-square test.

**Table 2:** Effect on Variables of Students’ and Knowledge-sharing partners groups at Repeated Time Measures.

		Main Effect ^a^			Interaction Effect ^a^		
		Time			Intervention × Time		
		F	*p*-Value	η_p_^2^	F	*p*-Value	η_p_^2^
Students							
Knowledge	Intervention	396.9	*p* < 0.001	0.72	8.4	*p* < 0.001	0.05
	Control						
Perceived susceptibility	Intervention	75.7	*p* < 0.001	0.33	55.5	*p* < 0.001	0.26
	Control						
Perceived severity	Intervention	28.0	*p* < 0.001	0.15	2.7	0.071	0.02
	Control						
Perceived benefits	Intervention	322.6	*p* < 0.001	0.68	19.0	*p* < 0.001	0.11
	Control						
Perceived barriers	Intervention	30.0	*p* < 0.001	0.16	28.0	*p* < 0.001	0.15
	Control						
Self-esteem	Intervention	107.2	*p* < 0.001	0.41	94.3	*p* < 0.001	0.38
	Control						
Healthy practice	Intervention	71.5	*p* < 0.001	0.32	26.1	*p* < 0.001	0.14
	Control						
**Knowledge-sharing partners**							
Perceived susceptibility	Intervention	28.33	*p* < 0.001	0.15	7.20	0.001	0.04
	Control						
Perceived severity	Intervention	27.59	*p* < 0.001	0.15	0.27	0.805	0.00
	Control						
Perceived barriers	Intervention	5.36	0.005	0.03	1.62	0.198	0.01
	Control						
Healthy practice	Intervention	34.32	*p* < 0.001	0.18	6.92	0.001	0.04
	Control						

Knowledge score: 0–14 (14 items); Health belief sub scales score: perceived susceptibility: 4–16 (4 items), perceived severity: 4–16 (4 items), perceived benefits: 6–24 (6 items), perceived barriers: 4–16 (4 items); Self- esteem score: 0–30 (10 items) Healthy practice score for students: 0–5 (exercise habit, sleeping hours, healthy diet, cancer talk, salt consumption); Healthy practice score for partners: 0–6 (Smoking habit, drink alcohol, exercise habit, healthy food consumption, care about salt intake, cancer screening test); ^a^ Two-way repeated measures ANOVA.

**Table 3:** Logistic regression on the change of healthy practice in 3-months among students.

Variables	Intervention Group	Control Group
	OR (95% CI)	OR (95% CI)
Exercise habit	3.16 (1.97–5.06) **	0.69 (0.42–1.12)
Sleeping time	1.71 (1.04–2.82) *	0.62 (0.40–0.98) *
Healthy diet consumption	2.76 (1.66–4.60) **	4.50 (2.76–7.35) **
Salt consumption control	1.41 (0.90–2.22)	1.19 (0.77–1.84)
Cancer talk with others	19.58 (10.50–36.52) **	1.06 (0.66–1.70)

* *p* < 0.05; ** *p* < 0.001; OR: Odds Ratio; CI: Confidence interval. Independent variable: Time.

**Table 4:** Logistic regression on the change of healthy practice in 3-months among knowledge-sharing partners.

Variables	Intervention Group	Control Group
	OR (95% CI)	OR (95% CI)
Smoking habit	1.00 (0.47–2.12)	0.79 (0.37–1.71)
Alcohol habit	2.27 (1.31–3.94) *	1.42 (0.88–2.29)
Exercise habit	5.33 (3.11–9.15) **	1.23 (0.65–2.34)
Healthy diet consumption	3.24 (1.73–6.08) **	2.96 (1.72–5.09) **
Salt consumption control	1.26 (0.79–2.01)	0.78 (0.50–1.21)
Cancer screening approach	4.06 (1.79–9.24) *	2.02 (0.36–11.22)

* *p* < 0.05; ** *p* < 0.001; OR: Odds Ratio; CI: Confidence interval. Independent variable: Time.

## Data Availability

The data presented in this study are available on request from the corresponding author. The data are not publicly available due to privacy.
